# Correlation between radiological and histopathological findings in patients undergoing nephrectomy for presumed renal cell carcinoma on computed tomography scan at Grey’s Hospital

**DOI:** 10.4102/sajr.v22i1.1339

**Published:** 2018-10-10

**Authors:** Nompumelelo E. Mlambo, Nondumiso N.M. Dlamini, Ronald J. Urry

**Affiliations:** 1 Department of Radiology, College of Health Sciences, University of KwaZulu-Natal, South Africa; 2 Department of Urology, College of Health Sciences, University of KwaZulu-Natal, South Africa

## Abstract

**Background:**

The incidence of renal cell carcinoma (RCC) is increasing globally owing to the increased use of cross-sectional imaging. Computed tomography (CT) scan is the modality of choice in the diagnosis and pre-operative assessment of RCC. Nephrectomy is the standard treatment for RCC and pre-surgery biopsy is not routinely practised. The accuracy of CT diagnosis and staging in a South African population has not been established.

**Objectives:**

To determine the accuracy of CT scan in the diagnosis and pre-operative staging of RCC at Grey’s Hospital.

**Methods:**

A retrospective chart review was performed; CT scan reports and histopathological results of adult patients who underwent nephrectomy for presumed RCC on CT scan between January 2010 and December 2016 were compared.

**Results:**

Fifty patients met the inclusion criteria for the study. CT significantly overestimated the size of renal masses by 0.7 cm (*p* = 0.045) on average. The positive predictive value of CT for RCC was 81%. Cystic tumours and those 4 cm and smaller were more likely to be benign. CT demonstrated good specificity for extra-renal extension, vascular invasion and lymph node involvement, but poor sensitivity.

**Conclusion:**

In our South African study population, CT is accurate at diagnosing RCC, but false-positives do occur. Non-enhancing or poorly enhancing, cystic, fat-containing and small lesions (4 cm or smaller) are more likely to be benign and ultrasound-guided biopsy should be considered to avoid unnecessary surgery. CT assessment of extra-renal extension and vascular invasion is challenging and additional imaging modalities such as magnetic resonance imaging (MRI) venogram, duplex Doppler ultrasound or Positron emission tomography–computed tomography (PET/CT) may be beneficial.

## Introduction

The prevalence of renal cell carcinoma (RCC) is increasing globally.^1^ This is partly owing to the increasing use of cross-sectional imaging in the clinical assessment of patients, leading to more tumours being discovered incidentally.^2,3^ Imaging plays a key role in the diagnosis of RCC and computed tomography (CT) scan is the modality of choice.^3,4^ Surgery is the standard treatment for non-metastatic RCC.^4,5^ Currently, in South Africa, pre-surgery biopsy of renal masses in adults is not routinely practised, and nephron sparing surgery is not routinely offered, particularly in the resource-constrained state sector.^5^ Accurate imaging is therefore essential, not only for establishing the likely diagnosis but also for staging, surgical planning, determining the extent of nephrectomy to be performed (radical or partial) and choosing the surgical approach.^1,2,3,4^

There are no data to determine the accuracy of CT imaging in the diagnosis and staging of RCC in South African patients. A better understanding of benign conditions presumed to be RCC on CT imaging and the accuracy of staging of renal tumours has the potential to reduce unnecessary nephrectomies and direct the use of pre-operative biopsy of renal masses.

## Methods

### Setting

Grey’s Hospital is a tertiary 530-bed academic hospital in Pietermaritzburg, South Africa. It has a wide catchment area covering a population of approximately 3 million people. It is the tertiary referral centre for urology and radiology for the entire referral area which includes primary health care clinics, district and regional hospitals.

### Data collection

All adult patients who underwent nephrectomy at Grey’s Hospital for presumed RCC on CT imaging from 01 January 2010 to 31 December 2016 were included in the study. A retrospective chart review was performed, and patient records were traced using theatre registers. Computed tomography reports and images were retrieved from the radiology information system (RIS) and picture and archiving system (PACS). Histopathological results were obtained from the National Health Laboratory Service (NHLS). Data collected included patient demographics, imaging findings and histopathological findings. Specifically, final histology diagnosis, tumour size, local invasion, vascular invasion and lymph node spread were assessed.

Computed tomography scan and histopathology reports produced during patient investigation and management were used, and images and specimens were not reviewed or re-reported for this study. All CT scan reports were overseen by a specialist radiologist during routine clinical reporting but not all by the same radiologist. Tumours were staged according to the American Joint Committee on Cancer tumour, nodes and metastases (TNM) classification.^6^ Cystic lesions were classified according to the Bosniak classification.^7^

### Computed tomography scanning methodology

All CT scans were performed using a Siemens Somatom Sensation Cardiac 64 slice scanner (Siemens Medical Solutions SW, Erlangen) with 0.5 s gantry rotation speed and a tube voltage of 120 KV. The tube current was determined using an automated current modulator. Scans were performed using collimation with slice thickness of 5 mm, pitch of 1.15 and image reconstruction of 1 mm. For contrast-enhanced scans, 100 ml of Omnipaque 300 contrast was injected intravenously at a flow rate of 3 ml/s.

Scans were performed in four phases. An unenhanced phase was used to provide a baseline to determine enhancement, and for assessment of the presence of intralesional calcifications and fat. An arterial phase (corticomedullary phase) at 10 s delay was used to enable the differentiation of an enhanced cortex and medulla and allow for the identification of renal vein tumour invasion. A parenchymal phase (porto-venous/nephrographic phase) at 70 s delay was used to identify and characterise small renal masses and assess for inferior vena cava (IVC) tumour invasion. An excretory phase at 10 min delay was used to delineate the relationship of the tumour to the collecting system. Patients were scanned from the lower thorax to the pubic symphysis in all phases except for the arterial phase where they were scanned from the diaphragm to the iliac crests. Table 1 highlights the main CT features that were used to differentiate benign from malignant renal tumours.

**TABLE 1:** Criteria for differentiating benign and malignant renal tumours on computed tomography scan.

CT features for benign renal tumours		CT features for malignant renal tumours	
Definitive	Probable	Definitive	Probable
Purely cystic with imperceptible walls	Solid and homogeneously enhancing	Heterogeneously enhancing solid mass	Complex cystic mass with enhancing solid component
Cystic with thin hairline septate	Ill-defined with perinephric fat stranding	Extra-renal extension, that is, renal vein tumour thrombus, capsular invasion	Largest diameter, ≥ 4 cm
Cystic with punctate calcifications	Largest diameter, < 4 cm	Renal mass with evidence of distant metastases	Solid mass with coarse calcifications
Macroscopic fat	Post IV contrast enhancement less than 20 HU	Post IV contrast enhancement greater than 20 HU	Ill-defined margins
Calcification conforming to collecting system	Well-defined margins	-	-

CT, computed tomography; IV, intravenous; HU, Hounsfield units.

### Statistical analysis

Data were collected and analysed using the IBM Statistical Package for Social Sciences (SPSS) version 24 (IBM Corp, Armonk, New York, The United States of America). The Pearson Chi-squared test (χ^2^) was used to compare categorical variables. If the projected frequency in a cell of a two-by-two table, assuming a null hypothesis, was less than five observations, the Fischer’s Exact test was used. The Student’s *t*-test was used to compare quantitative variables. A *p*-value of less than 0.05 (5%) was considered statistically significant. The sensitivity, specificity, positive predictive value (PPV) and negative predictive value (NPV) of CT scan for variables studied were determined using the histopathological results as the gold standard.

## Ethical considerations

This study was a retrospective chart review study. Ethics approval for this study was granted by the Biomedical Research Ethics Committee (BREC) of the University of KwaZulu-Natal (BE004/17).

## Results

Of the 119 adult patients who underwent nephrectomy during the study period, 35 were excluded because their CT scan images were not available, 26 because the indication for nephrectomy was not suspected to be RCC, 7 because the histopathological results could not be traced and 1 for being under the age of 18 years. Fifty patients fulfilled the inclusion criteria. The mean age was 54 years (range 23–74 years) with a male to female ratio of 1:1.

Thirteen patients (26%) had incidentally discovered renal lesions picked up on other imaging modalities or on CT performed for non-related pathology, 34 patients (68%) were symptomatic with an abdominal mass, flank pain or haematuria and four patients (8%) had imaging to identify a primary lesion after discovery of metastatic disease.

**FIGURE 1:**
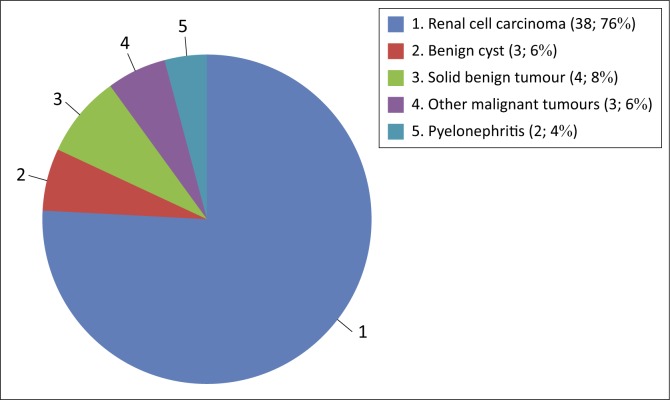
Distribution of histopathological findings: Histology final diagnosis (*n*; %)

The mean tumour size was 9 cm (range 1 cm–28 cm). Histopathology findings are illustrated in Figure 1. The non-RCC malignant tumours found at histopathology were adenocarcinoma, transitional cell carcinoma and squamous cell carcinoma. The benign lesions found were solid benign tumours (angiomyolipoma, oncocytoma and leiomyoma) and benign cysts. In two patients, inflammatory changes in the kidney were mistaken for RCC on imaging.

Table 2 demonstrates a comparison of patient and tumour characteristics on imaging in patients with confirmed RCC (*n* = 38) and those with benign findings on histology (*n* = 9).

**TABLE 2:** Comparison of patient and tumour characteristics on imaging in patients with confirmed renal cell carcinoma and those with benign findings on histology (excluding the three patients with non-renal cell carcinoma malignancy).

Variables	RCC (*n* = 38)		Benign pathology (*n* = 9)	
Characteristics	*N*	%	*N*	%
**Age group**				
< 40 years	4	11	2	22
40–59 years	18	47	4	44
60–79 years	16	42	3	33
**Total**	**38**	**-**	**9**	**-**
**Gender**				
Male	19	50	4	44
Female	19	50	5	56
**Total**	**38**	**-**	**9**	**-**
**CT tumour characteristics**				
Solid mass	35	92	3	33
Bosniak 3 lesion	1	3	2	22
Bosniak 4 lesion	2	5	4	44
**Total**	**38**	**-**	**9**	**-**
**CT tumour size**				
≤ 4	8	21	6	67
4–6	3	8	2	22
6–9	7	18	0	0
≥ 10	20	53	1	11
**Total**	**38**	**-**	**9**	**-**

CT, computed tomography; RCC, renal cell carcinoma.

**FIGURE 2:**
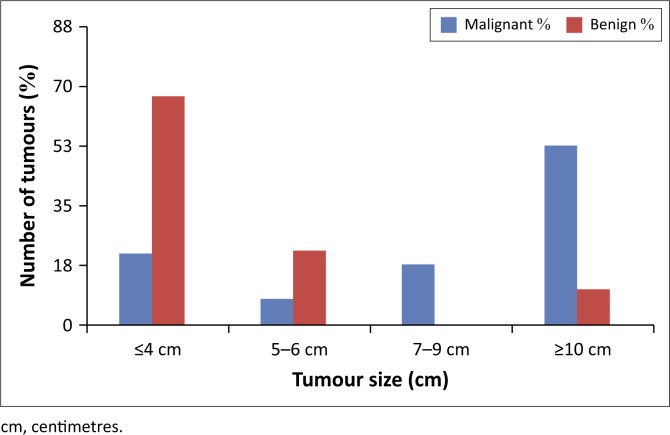
Comparison of computed tomography tumour size between renal cell carcinomas and benign lesions.

The single benign tumour greater than 10 cm was a fat-poor angiomyolipoma which was reported on CT as being a possible RCC, illustrated in Figure 7. Of the cystic lesions, 66% were benign. Figure 2 illustrates the CT size of RCCs compared to benign tumours. The majority of benign lesions (67%) were less than 4 cm on imaging, and CT imaging size > 4 cm was significantly associated with RCC (*p* = 0.002). Patients younger than 40 years were not significantly more likely to have benign lesions than older patients.

Computed tomography significantly overestimated RCC tumour size compared to measured size at histopathology, with a mean overestimation of 0.7 cm (*p* = 0.046). As a result, there was post-operative reduction in the T-stage in six patients (16%). Computed tomography demonstrated a PPV of 81% for the diagnosis of RCC. The sensitivity, specificity, PPV and NPV of CT for tumour staging are tabulated in Table 3. Very few patients had positive findings for local invasion, vascular invasion and lymph node involvement, resulting in high levels of agreement between CT and histopathology findings.

**TABLE 3:** Sensitivity, specificity, positive predictive value and negative predictive value of computed tomography scan for tumour staging.

Parameter	Sensitivity (%)	Specificity (%)	PPV (%)	NPV (%)
Local invasion	38	89	50	83
Vascular invasion	20	97	50	88
Lymph node involvement	83	87	56	96

PPV, positive predictive value; NPV, negative predictive value.

**FIGURE 3:**
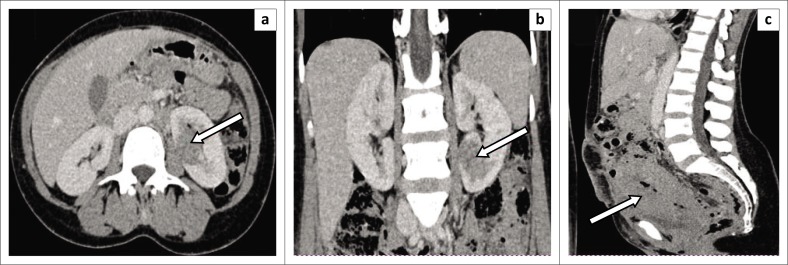
Incidental finding of renal cell carcinoma (RCC) on a post-caesarean section ultrasound. Contrast-enhanced portovenous phase computed tomography images show: (a) an ill-defined hypoenhancing mass lesion in the left renal cortex (arrow) with a poor plane of separation between the mass and the psoas muscle, concerning for extra-renal extension. (b) Coronal image demonstrates that the mass arises in the lower pole of the left kidney. (c) Sagittal image demonstrates post-partum changes with an enlarged uterus as well as a thickened uterine wall and air within the uterine cavity (arrow). A diagnosis of clear cell RCC was confirmed at histology.

**FIGURE 4:**
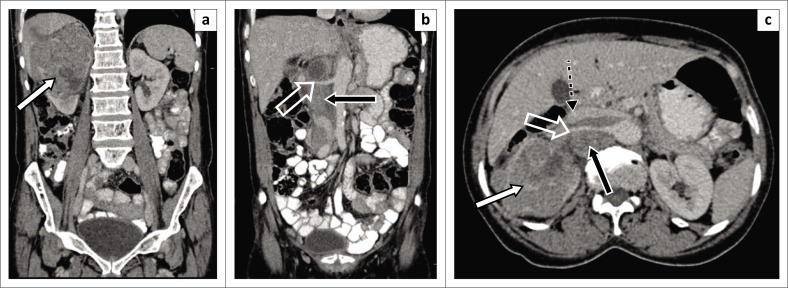
Coronal (a, b) and axial (c) contrast-enhanced portovenous phase computed tomography scan images demonstrate a large right renal mass with capsular rupture and tumour extension into the perinephric space (white arrow). Extensive metastatic retroperitoneal lymphadenopathy (black arrow) with encasement and subsequent narrowing of the right renal artery (open white arrow). There is also compression and anterior displacement of the inferior vena cava (IVC) (dashed black arrow). There was no tumour thrombus in the renal vein (not shown) and IVC. The findings of local tumour invasion involving the perinephric space and lymph node spread were confirmed at histology.

**FIGURE 5:**
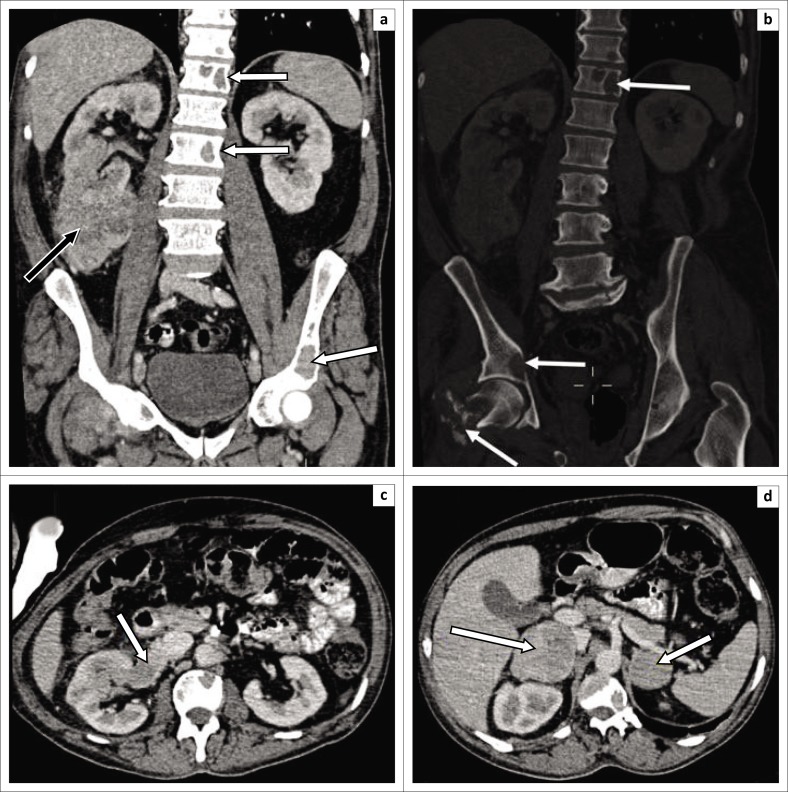
Renal cell carcinoma (RCC) in a patient who presented with a pathological right femoral neck fracture. Portovenous phase computed tomography scan coronal images demonstrate (a) a large exophytic heterogeneously enhancing right renal lower pole mass (black arrow) with perinephric fat stranding and thickening of Gerota’s fascia. (a and b) Multiple lytic skeletal metastases in the spine, pelvis and right femur with associated neck of femur fracture (white arrows). (c) An enhancing tumour thrombus in the right renal vein (arrow). There is no extension of the tumour thrombus to the inferior vena cava. (d) Bilateral adrenal gland metastases (arrows) were present. These findings are consistent with stage 4 disease.

**FIGURE 6:**
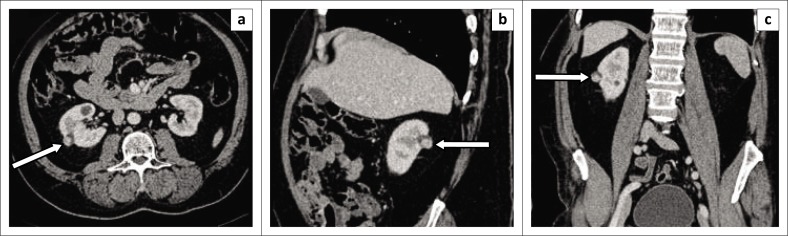
Non-renal cell carcinoma (RCC) benign renal tumour. Portovenous phase-enhanced computed tomography (CT) scan (a) axial, (b) sagittal and (c) coronal demonstrates a small exophytic rounded cystic lesion with a large uniformly enhancing solid component in the right renal mid pole (white arrow). No associated fat stranding. This lesion was assessed as RCC at CT scan. Post-nephrectomy histology results showed a benign leiomyoma.

**FIGURE 7:**
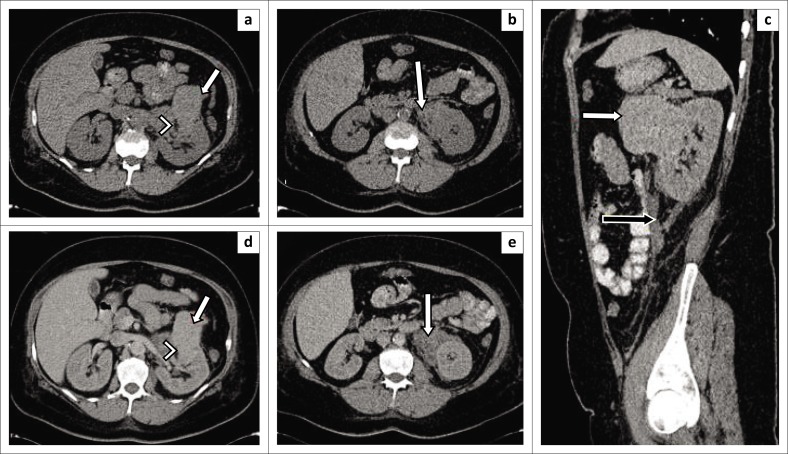
(a, b) Non-enhanced axial computed tomography (CT) images show a large exophytic high-density soft tissue mass arising from the left kidney mid pole anteriorly (arrow in image a), perinephric haemorrhage and fat stranding (white arrow in image b). Sagittal (c) – There is also thickening of the Gerota’s fascia (black arrow). Portovenous phase axial images (d) and (e) demonstrate mild enhancement of the renal mass and the perinephric collections. A small focus of intralesional fat (arrowhead) is demonstrated by an arrowhead in the image (a, d). This was diagnosed as haemorrhagic renal cell carcinoma on CT scan, and histology results showed angiomyolipoma (lipid-poor).

**FIGURE 8:**
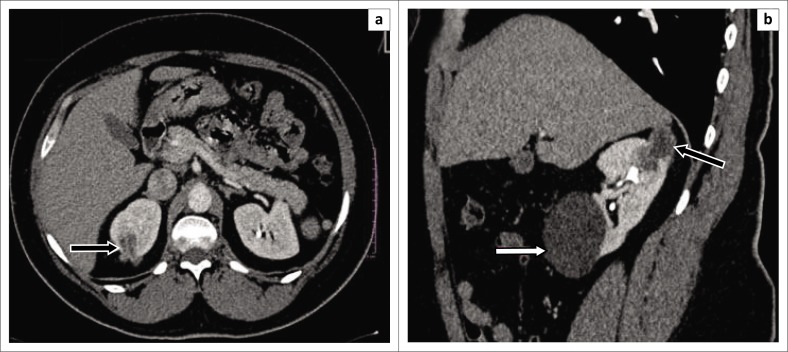
Nephrographic phase computed tomography images (a) axial and (b) sagittal demonstrate a multiloculated exophytic complex cystic mass with rim enhancement and enhancing thick septae in the right renal upper pole (black arrow). No associated perirenal fat stranding. Computed tomography diagnosis of cystic renal cell carcinoma was made; histological diagnosis was that of a simple cyst. A further large simple renal cyst is noted in the lower pole of the right kidney (white arrow).

Figures 3–5 demonstrate true-positive findings in three patients with confirmed RCC on histopathology, and Figures 6–8 demonstrate false-positive findings in three patients with suspected RCC on imaging but in whom leiomyoma, angiomyolipoma and a benign cyst were diagnosed at histopathology.

Most patients underwent radical nephrectomy. Of the 44 (88%) patients who underwent radical nephrectomy, 89% had RCC and 11% had benign pathology. The mean tumour size on imaging in these patients was 9.5 cm. Six patients (12%) underwent partial nephrectomy for lesions on CT scan of less than 4 cm. Of these, 33% had RCC and 67% had benign pathology. The mean tumour size on imaging in these patients was 3.3 cm.

## Discussion

Computed tomography is widely accepted as the diagnostic modality of choice in the diagnosis and staging of RCC. It has a diagnostic accuracy of up to 93% and sensitivity and specificity for staging of up to 90%.^1,2,8^

Our study demonstrated a PPV of 81% and a false-positive rate of 19% for CT diagnosis of RCC. In two studies of patients who underwent surgery for presumed RCC on CT, Kutikov et al. found a benign rate of 16.1%^9^ and Silver et al. of 16.9%.^10^ In a similar study, Fuji et al. found a benign histopathological diagnosis in 11% of the partial nephrectomy specimens and 3.5% of radical nephrectomy specimens.^11^ Compared to these studies, our study demonstrates a slightly higher rate of false-positive results. In contrast, Alkaabnah et al. reported a higher benign rate of 30% in patients undergoing partial nephrectomy.^12^ Nakashima et al. attributed a low benign incidence rate of 6.63% in their study to the use of magnetic resonance imaging (MRI) in conjunction with CT scan in their patients, as MRI is known to be more sensitive in detecting small amounts of intralesional fat.^8^

Computed tomography scanning is known to overestimate the tumour size compared to pathological size which may lead to tumours being down-staged post-surgery.^2,3^ Tumour size plays an important role in surgical planning because tumours smaller than 4 cm are likely to be amenable to partial nephrectomy, whereas larger tumours will likely require radical nephrectomy.^3,13^ Partial nephrectomy is further favoured in tumours with a peripheral location in the kidney and is specifically indicated in patients with an absent contralateral kidney, bilateral kidney tumours, renal insufficiency and risk factors for future renal impairment.^4,10,11,13^ We found that CT significantly overestimated tumour size by 0.7 cm on average and that this resulted in down-staging in some patients. Chen et al. also demonstrated tumour size overestimation by CT. This was seen particularly in tumours less than 7 cm.^2^ Early arterial clamping during nephrectomy may result in a decrease in tumour blood volume. This is thought to be the main reason for the smaller pathological tumour size compared to radiological size, as most RCCs are hypervascular.^1,3^ Another reason suggested is that tumour fixation with formalin may cause shrinkage of the tumour.^1,2^

Extra-renal tumour extension is assessed on CT by evaluation of the integrity of the renal capsule, the presence of perinephric fat stranding and the presence of enhancing nodules.^3^ Bradley et al. demonstrated that the presence of perinephric collateral vessels and thickening of Gerota’s fascia are more reliable indicators of perinephric tumour extension than the presence of fat stranding alone.^14^ There are other causes of perinephric fat stranding which include oedema, fibrosis, vascular engorgement and inflammation secondary to renal calculi or infection.^4^

Computed tomography tends to overdiagnose lymph node spread, which is defined as a lymph node with short axis diameter of greater than 1 cm.^3^ This is a poor indicator, however, because nodal enlargement can result from reactive hyperplasia owing to current or previous inflammation. Also, small lymph nodes can harbour micro-metastases, resulting in false-negative findings.^3,13^ Lymph nodal invasion can be differentiated from reactive hyperplasia by assessing the enhancement pattern as metastatic lymph nodes tend to have similar enhancement pattern to the primary tumour.^4^

Assessment of the renal veins and IVC for vascular invasion is performed on images obtained during corticomedullary and nephrographic phases, respectively.^4,13^ Maximal opacification of the renal vessels to allow for confident diagnosis of tumour extension into the renal vein is achieved in the late corticomedullary phase (25–70 s post-intravenous contrast administration). Vascular invasion is indicated by the presence of an intra-vascular enhancing filling defect, focal venous wall enhancement and infiltration of adjacent soft tissues. On rare occasions, tumour thrombus extends into the right atrium and pulmonary arteries.^3,4^ Adequate assessment of tumour thrombus extent is crucial for patient counselling and surgical planning. A thoracoabdominal surgical approach is required for tumour thrombus extending into the supra-hepatic IVC.^1,3^

In our study, CT showed high specificity but unexpectedly poor sensitivity for extra-renal extension and venous invasion, suggesting that CT is not effective at identifying these findings. This is contradicted by what is described in the literature where CT has been well documented to have high sensitivity and specificity.^1,2,8^ We believe that this is partly explained by the low number of patients with extra-renal extension and venous invasion in the study. Further scrutiny, however, may support the concept that CT diagnosis of extra-renal and vascular invasion in RCC is difficult. Sokhi et al. reported CT sensitivity for renal vein invasion in T3a RCC to be 59% – 69%.^15^ Computed tomography is unlikely to perform well in cases of microscopic or small volume extension into the perinephric tissue and renal vein. This highlights the importance of using indirect signs such as perinephric fat stranding, thickening of Gerota’s fascia and collateral vessels. If there is doubt about vascular invasion, an MRI venogram or a duplex Doppler ultrasound may be beneficial.^16^

Fluorodeoxyglucose (FDG) PET/CT imaging has a limited role in imaging of primary renal tumours owing to physiological activity in the urinary system. However, PET and/or CT has a role in detecting tumour spread and is more sensitive for skeletal metastases than bone scintigraphy. PET and/or CT can be used to differentiate between tumour thrombus and bland thrombus. Assessing lymph node spread remains challenging as PET and/or CT has reduced sensitivity in identifying tumour spread in lymph nodes less than 7 mm owing to reduced spatial resolution. Furthermore, PET and/or CT demonstrates increased radiotracer uptake in both reactive lymphadenopathy and metastatic lymph nodes, resulting in a potential for false-positive results.^17^

The most common benign tumour presumed to be RCC on CT in our study was a complex cystic mass which was classified as either Bosniak 3 or Bosniak 4. Figure 8 demonstrates a cystic lesion graded as a Bosniak 3 cyst on CT scan, which was found not to be a malignancy on histopathology. Magnetic resonance imaging has been suggested to better evaluate Bosniak 3 lesions. However, it tends to exaggerate septal thickness and demonstrates variable enhancement owing to variation in image quality, risking an overestimation of the allocated Bosniak grade.^18^ Despite any additional diagnostic value it may add, MRI may not be feasible as it is expensive and not readily available.

Other benign lesions discovered in our study were oncocytoma, benign leiomyoma, angiomyolipoma and pyelonephritis with or without nephrolithiasis. Some studies have reported lipid-poor angiomyolipoma to be the most common benign tumour presumed to be RCC on CT scan, followed by oncocytoma.^8,9,10,11,16^ Ethnicity may predict the type of benign tumours found at nephrectomy performed for presumed RCC, with most Asian studies reporting angiomyolipoma as the most common benign tumour and studies from the US reporting oncocytoma.^2,8,10,11,12^ Oncocytoma cannot be confidently distinguished from RCC on imaging.^11,16^ We expected that given the high burden of infectious diseases in South Africa, we would see more inflammatory lesions such as xanthogranulomatous pyelonephritis, tuberculosis and aspergillosis being mistaken for RCC. Our study focused on patients with an imaging diagnosis of RCC, implying that as these conditions were not found in our study, they are not being mistaken for RCC.

It is well established that small renal tumours are more likely to be benign, with a 1.33 times increased risk of malignancy per centimetre increase in the size of a tumour.^19^ This is in line with our finding that the majority of benign lesions were less than 4 cm and that there was a significant association between larger tumours and RCC. Until recently, pre-surgery biopsy of renal masses has not been widely practised because of concerns of tumour seeding and lack of therapeutic benefit. This mindset is changing, however, with recent evidence coming out in favour of pre-surgery biopsy of small renal masses to reduce overall morbidity and avoid over-treatment of benign lesions.^20^ We found, further, that smaller lesions were more likely to be treated by partial nephrectomy.

This study was performed in a single centre and is limited by the small sample size, which impacted the assessment of the sensitivity and specificity of CT scan for staging of RCC. A multicentre study performed in a larger cohort may yield more accurate results. Furthermore, false-negative results could not be assessed in our study because we only included patients with the pre-operative radiological diagnosis of RCC.

## Conclusion

Computed tomography scan is the modality of choice for diagnosis and staging of RCC. In our population, CT is accurate at diagnosing RCC. False-positives occur in the cases of benign cystic lesions, benign solid tumours and inflammatory lesions. The high burden of infectious disease in South Africa does not appear to increase the false-positive rate. Computed tomography overestimates the size of renal lesions, which may result in down-staging at histopathology. Non-enhancing or poorly homogeneously enhancing masses, cystic lesions, especially those with simple features, fat-containing lesions and lesions 4 cm or smaller are more likely to be benign. In these cases, CT, MRI or ultrasound-guided biopsy should be considered to avoid unnecessary surgery. Computed tomography assessment of extra-renal extension and vascular invasion is challenging and additional imaging modalities such as MRI venogram, duplex Doppler ultrasound or PET and/or CT may be beneficial.
